# The use of labelled leucocyte scintigraphy to evaluate chronic periprosthetic joint infections: a retrospective multicentre study on 168 patients

**DOI:** 10.1007/s10096-019-03587-y

**Published:** 2019-06-20

**Authors:** Philippe Blanc, Eric Bonnet, Gérard Giordano, Jacques Monteil, Anne-Sophie Salabert, Pierre Payoux

**Affiliations:** 1 Department of Nuclear Medicine, Pierre-Paul Riquet Hospital, CHU Purpan, Place du Dr Baylac, TSA 40 031, 31059 Toulouse Cedex 9, France; 2 Department of Nuclear Medicine, Dupuytren University Hospital, 2 avenue Martin Luther King, 87042 Limoges Cedex, France; 3 Mobile Team of Infectious Disease, Joseph Ducuing Hospital, 15, Rue de Varsovie, 31027 Toulouse Cedex 3, France; 4 Orthopedic Surgery and Traumatology, Joseph Ducuing Hospital, 15, Rue de Varsovie, 31027 Toulouse Cedex 3, France; 5 ToNIC, Toulouse NeuroImaging Centre, CHU Purpan – Pavillon Baudot, Place du Dr Baylac, 31024 Toulouse Cedex 3, France

**Keywords:** Scintigraphy, Infection, Prosthesis, Radiolabelled leucocytes, Pathogens, Antibiotics

## Abstract

Labelled leucocyte scintigraphy (LS) is regarded as helpful when exploring bone and joint infections. The aim of this study was to evaluate the utility of LS for the diagnosis of chronic periprosthetic joint infections (PJIs) in patients exhibiting arthroplastic loosening. One hundred sixty-eight patients were referred to centres for treatment of complex PJI. One hundred fifty underwent LS using ^99m^Tc-HMPAO (LLS); 18 also underwent anti-granulocyte scintigraphy (AGS) and 13 additional SPECT with tomodensitometry imaging (SPECT-CT). The LS results were compared with bone scan data. For all, the final diagnoses were determined microbiologically; perioperative samples were cultured. LS values were examined, as well as sensitivity by microorganism, anatomical sites, and injected activity. LS results were also evaluated according to the current use of antibiotics or not. The sensitivity, specificity, and positive predictive value of LLS were 72%, 60%, and 80%, respectively. LLS performed better than did AGS. SPECT-CT revealed the accurate locations of infections. The sensitivity of LS was not significantly affected by the causative pathogen or the injected activity. No correlation was evident between the current antibiotic treatment and the LS value. The test was more sensitive for knee (84%) than hip arthroplasty (57%) but was less specific for knee (52% vs. 75%). Sensitivity and specificity of LLS varied by the location of infection bone scan provide no additional value in PJI diagnosis. Current antibiotic treatment seems to have no influence on LS sensitivity as well as labelling leukocyte activity or pathogens responsible for chronic PJI.

## Introduction

As life expectancy increases, total hip and knee joint replacements are becoming more common; these procedures improve quality-of-life. Chronic periprosthetic joint infection (PJI) is a serious complication of joint surgery causing substantial morbidity (chronic pain, loss of ambulation) and requiring complex treatment (repeat surgery, long-term antibiotic therapy). Moreover, the financial costs are high, despite the low frequencies of complications (which arise after 1% of total hip arthroplasties [THAs] and 2% of total knee arthroplasties [TKAs]) [1].

When the clinical signs of infection are not obvious, it can be very challenging to distinguish delayed infection from mechanical loosening. Use of the C-reactive protein level lacks specificity (Sp) and sensitivity (Se). Joint aspiration data are also unreliable in cases of chronic infection [2, 3]. Plain radiographs cannot differentiate septic from aseptic loosening, and artefacts frequently limit the utility of computed tomography and magnetic resonance imaging.

Radionuclide imaging, which is not affected by metallic hardware, can be helpful when exploring bone and joint infections. In vitro labelled autologous leucocyte scintigraphy (LLS) is a valuable imaging modality [4]. Other radiopharmaceuticals (^18^FDG, ^18^F-fluoride, radiolabelled peptides, and radiolabelled antibiotics) have also been used but afford no significant advantage over LLS or anti-granulocyte scintigraphy (AGS) when evaluating PJI [4–6].

The aim of the present study was to evaluate the usefulness of leucocyte scintigraphy ([LS], meaning LLS and AGS) in relation with the pathogens involved in PJI. We explored the detection rates of complex chronic bone and joint infections in two referral centres and evaluated the effects of technical factors on radionuclide imaging data.

## Patients and methods

### Patient population

Between June 2007 and April 2014, 168 consecutive patients with mechanical joint loosening from two referral centres treating complex bone and joint infections were enrolled retrospectively. Only patients who had undergone operations at least 6 months prior to scintigraphy were included. All arthroplasties were revised because of loosening of the prostheses. A total of 37 patients with a suspicion of chronic PJI were prescribed antibiotics at the time of scintigraphy. The final diagnoses relied exclusively on microbiological data from perioperative samples. Infection was confirmed when at least two perioperative samples were positive for the same bacterial or fungal strain [6].

To limit potentially confusing factors, we excluded patients with periprosthetic fractures, inflammatory rheumatism, autoimmune disease, and scalable neoplasms and those undergoing immunosuppressive treatment. We also excluded those for whom only joint aspiration data (no surgical samples) were available.

**Fig. 1:**
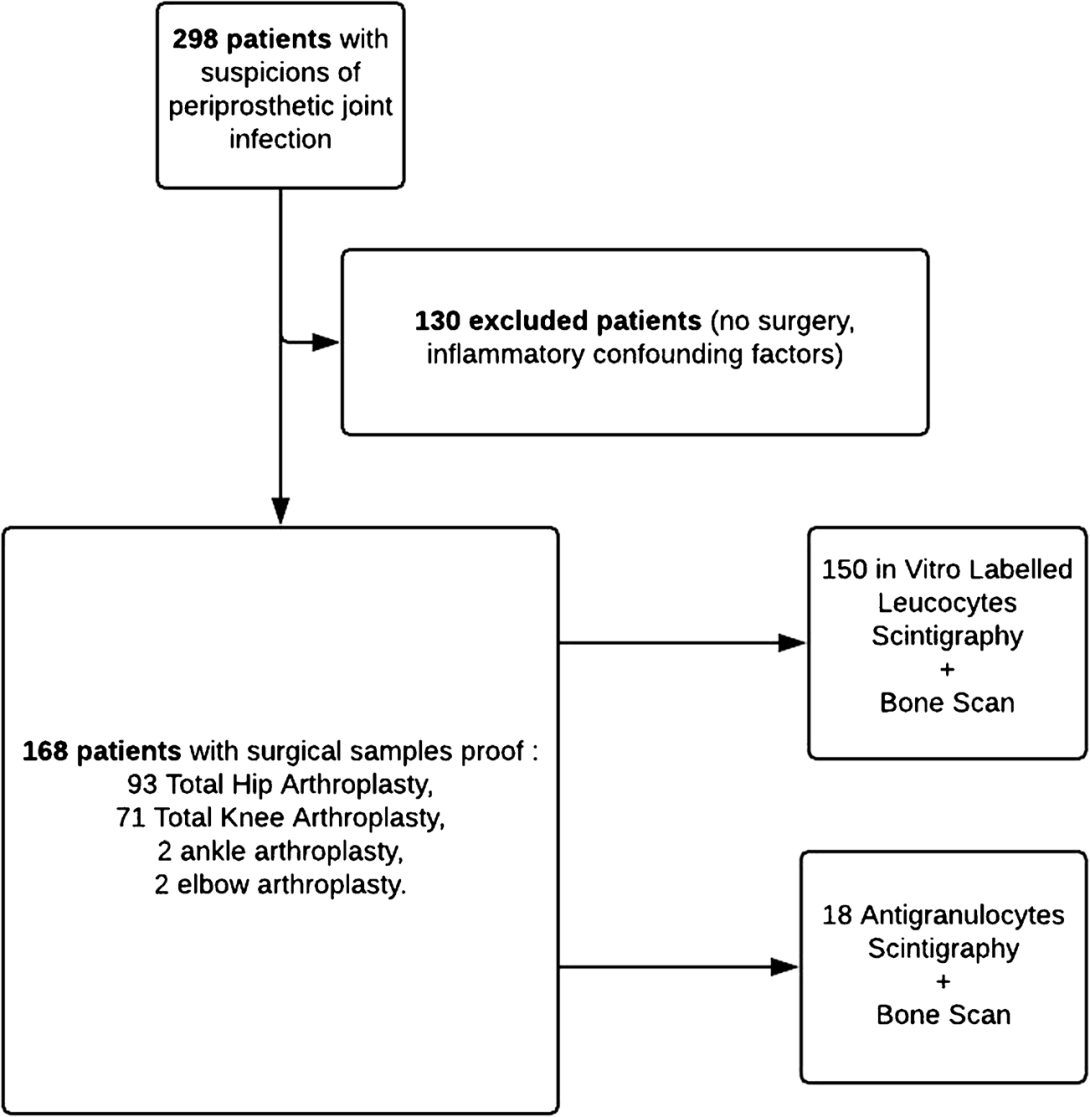
Flowchart of the study

All patients were evaluated in terms of clinical signs, medical history, and levels of biochemical markers (C-reactive protein and the erythrocyte sedimentation rate [ESR]) relevant to chronic PJI. Figure 1 shows a flow chart of the work. A total of 150 patients underwent ^99m^Tc-hexamethylpropyleneamine oxime (^99m^Tc-HMPAO)-labelled leucocyte scintigraphy (LLS), and 18 underwent anti-granulocyte scintigraphy (AGS). Of the latter patients, 13 underwent additional single-photon emission computed tomography with tomodensitometry imaging (SPECT-CT). The results of LLS and AGS were compared with those of whole-body bone scans (BSs). AGS was preferred to LLS if the ESR was less than 5 mm/h or the number of polynuclear leucocytes < 2000/mm^3^.

### Imaging protocol and analysis

In vitro labelled autologous leukocyte scintigraphy (LLS): leucocytes were obtained from 50-mL venous blood and labelled with 740–850 MBq ^99m^Tc-HMPAO (Ceretec®, GE Healthcare SA, France) according to the guidelines of the European Association of Nuclear Medicine [7]. After labelling, we re-injected cells carrying 99–824 MBq of the radiopharmaceutical. Planar images (both anterior and posterior views) of the joint were acquired at 4 h (early) and 24 h (late) after injection. For the early images, the acquisition time was 15 min using a high-resolution/low-energy collimator. For the late images, the acquisition time was increased to 25 min, because the count rate was low. Additional SPECT-CT imaging was sometimes performed instead of early planar acquisition (*n* = 7 patients) using a dual-head gamma camera equipped with a low-dose X-ray system.

Tracer distribution was analysed visually. An increase in uptake from 4 to 24 h was considered to indicate an infection. The absence of uptake was considered to reflect the absence of infection. A constant or decreasing uptake was considered to indicate a sterile inflammatory process rather than septic progression. SPECT-CT was considered useful when the site of infection was precisely located and/or when separate bone and soft tissue involvements were evident.

Anti-granulocyte scintigraphy (AGS) was performed after intravenous injection of 750–925 MBq ^99m^Tc-sulesomab (LeukoScan®, Immunomedics GmbH, Germany). Images were acquired 6 and 24 h after injection. The acquisition protocol and the mode of image analysis were similar to those of LLS. Additional SPECT-CT was performed on six patients.

BSs were performed 1 week before LLS to confirm loosening of the prosthesis. Patients underwent LLS if loosening was obvious on X-ray or computed tomography. Images were acquired 2 h after intravenous injection of ^99m^Tc-hydoxymethylene diphosphonate (^99m^Tc-HDP) (555 MBq for a 70-kg adult). Planar images were obtained in both the anterior and posterior views using a high-resolution parallel collimator. A BS was considered to indicate loosening when periprosthetic uptake was apparent 12 months after surgery and 24 months after TKA surgery (these intervals limited surgery-related artefacts). Infection was considered absent if no loosening was apparent.

All scintigraphic data were evaluated by two experienced nuclear physicians.

### Statistical analysis

The Se, Sp, positive predictive value (PPV), negative predictive value (NPV), accuracy, and statistical strength were calculated using classical methods and compared using Fisher’s test. Microbiological evaluation of surgical samples was the gold standard. Receiver-operator characteristic curve was realized. Rank correlations between the levels of injected radioactivity and the Se and Sp values were calculated using Spearman’s non-parametric test. The significance threshold was set at 0.05 (*p* < 0.05) and the threshold for statistical strength at 80%. SAS Enterprise Guide version 5.1 (SAS Institute, Cary, NC, USA) and MedCalc 13.1 (MedCalc Software, 8400 Ostend Belgium) were used for all statistical analyses.

## Results

The cohort parameters (prosthesis type, proportion of infected prostheses, mean age, and sex ratio) were statistically comparable between the two groups (Table 1). We evaluated 87 males and 81 females (mean age, 67 years). A total of 112 patients were infected, and 56 were sterile.

**Table 1:** Prosthesis group composition

	THA	TKA	EA	AA
Infected prosthesis				
Unit 1	26	11	0	0
Unit 2	36	37	0	2
Total	62	48	0	2
Non infected prosthesis				
Unit 1	15	9	0	0
Unit 2	16	14	2	0
Total	31	23	2	0

*THA* total joint arthroplasty, *TKA* total knee arthroplasty, *EA* elbow arthroplasty, *AA* ankle arthroplasty

### Overall performance

**Fig. 2:**
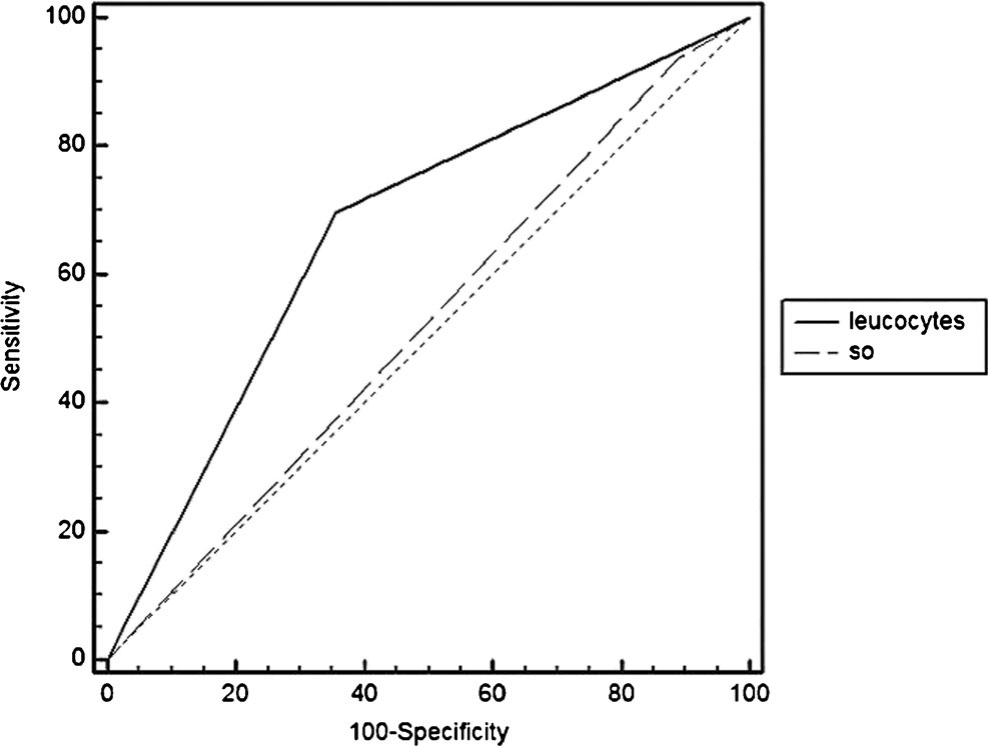
Comparative ROC curves of labelled leucocyte scintigraphy (LLS + AGS) and BS. Bone scan ROC curve (dashed line) was near to the random classification line. It was unable to distinguish septic from aseptic loosening. SO bone scan, Leucocytes leucocyte scintigraphy

Table 2 lists the Se, Sp, PPV, and accuracy of LLS, AGS, and BS. Neither the Se nor Sp differed significantly among the various modalities. Septic and aseptic loosening could not be distinguished on BSs (Fig. 2). The NPVs were relatively low.

**Table 2:** Overall performances of LLS, AGS and BS

		Nb	Sensitivity (%)	Specificity (%)	PPV (%)	NPV (%)	Accuracy (%)
LLS	Unit 1	43	72	64	81	53	70
	Unit 2	107	71,6	57,6	79	45	66
	Total	150	72	60	80	47	67
AGS	Unit 1	18	25	90	67	60	61
LLS + AGS		168	68	65	79	50	67
BS		124	94	11	65	50	64

*Nb* patients number, *PPV* positive predictive value, *NPV* negative predictive value, *LLS* in vitro labelled leucocyte scintigraphy, *AGS* anti-granulocyte scintigraphy, *BS* bone scan

Thirteen patients underwent SPECT-CT, the Se and Sp of which were 67% and 80%, respectively. No patient exhibited leucocyte accumulation in soft tissue.

### Influences of the prosthesis type, pathogen, antibiotic treatment, and injected activity

LS was more sensitive in TKA than THA patients (84% vs. 57%, *p* = 0.0002) but less specific in the former than the latter patients (52% and 75%, respectively; *p* = 0.0017). Only one of the two infected ankle prostheses was detected. Only one of the two aseptic loosenings of an elbow prosthesis was diagnosed correctly.

**Fig. 3:**
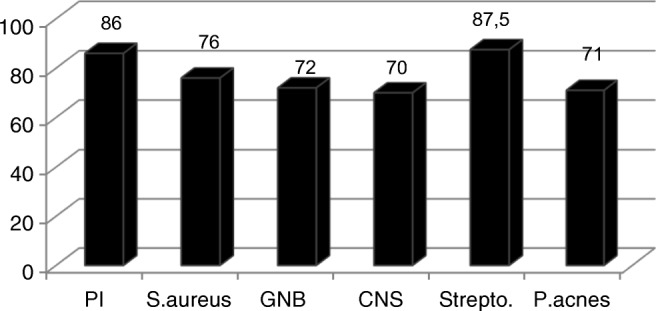
LLS sensitivity (in %) depending on pathogens. None of the results were statistically different (*p* > 0.05). PI polymicrobial infection, S.aureus *Staphylococcus aureus*, GNB Gram-negative bacilli, CNS coagulase negative staphylococci, Strepto. Streptococcus, P.acnes *Propionibacterium acnes*

Figure 3 shows the LS sensitivities according to the involved pathogen. No significant difference was apparent (*p* > 0.05). However, the statistical strength was < 80% and was thus inadequate to allow valid observations.

Thirty-seven patients were on antibiotics at the time of scintigraphy. No correlation was found between the current antibiotic treatment and LS data (*p* > 0.05). The LS Se and Sp were 76% and 63% for patients on antibiotics and 66% and 65% for those not on antibiotics, respectively. Seven false-negatives were found among those on antibiotics versus 29 among those not on antibiotics. Although no significant difference was evident between the two groups (*p* > 0.05), the statistical strength was < 20%.

We defined seven activity ranges of labelled white blood cell (WBC) (from < 200 to > 700 MBq). The number of patients in each range was similar [8–11]. Neither the Se nor Sp differed significantly among those exhibiting different activity ranges. We were unable to define a minimal extent of labelling affording optimal performance.

## Discussion

Microbiological and histological analyses of periprosthetic tissues are more useful than synovial fluid aspiration or assays of inflammatory blood markers when diagnosing chronic PJI [6, 7, 12]. We compared scintigraphic data with those from perioperative samples, to appropriately evaluate the utility of scintigraphy in chronic PJI diagnosis. Patients treated in the two referral centres were comparable in terms of sex ratio and mean age.

Many nuclear imaging studies have been published on PJI; significant differences in Se and Sp were apparent. Table 3 lists the features of a few studies published between 2000 and 2016. They included patients with hip and knee prostheses. The radiopharmaceuticals used were the same as those employed by us.

**Table 3:** Literature review of labelled leukocytes scintigraphy value

	Tracers	Number of prostheses	Sensitivity (%)	Specificity (%)
Simonsen et al. [13]	LLS	76	81	94
Teller et al. [14]	LLS	166	64	78
Larikka et al. [15]	LLS	30	62	100
Pelosi et al. [16]	LLS	95	85	71
Trevail et al. [17]	LLS	221	80	99.5
Erba et al. [18]	LLS	132	93	100
Gratz et al. [19]	AGS	20	100	83
Sousa et al. [20]	AGS	27	100	20
Rubello et al.(21)	AGS	78	93	78
Pakos et al.(22)	AGS	19	75	86

*LLS* in vitro labelled leucocyte scintigraphy, *AGS* anti-granulocyte scintigraphy

Various interpretive criteria were used in earlier studies [13, 14, 16–18, 23]. Some were qualitative in nature (visual comparisons of early and late acquisitions, comparisons between periprosthetic uptake and that of the surrounding bony tissue or the contralateral site) [13, 18]. Other authors employed semi-quantitative criteria (labelled WBC activity in the region of the symptomatic prosthesis, compared with bone marrow uptake) [24]. Various gold standards have been used to confirm diagnoses. Some authors have employed combined criteria (data on surgical samples with favourable clinical follow-up [25] or joint aspiration data with clinical assessment [8]).

The acquisition protocols differed greatly in previous publications. In some studies, early and late images were acquired over fixed times or to fixed numbers of counts. Usually, no corrections for isotope decay were made; the data were thus affected by variations in background activity. Erba et al. obtained good results by acquiring images at 3–4 h and 20–24 h in time mode with acquisition times corrected—24 h and correcting the data for isotope decay [13].

In terms of the Sp and Se of functional imaging, labelled WBC accumulation is considered to reflect inflammation rather than infection. We found that LLS had a Sp of 60%, slightly lower than the Sps reported in most previous studies (cf. Table 3). However, all of our patients had inflammatory conditions caused by prosthesis loosening; this reduced the Sp. The eight most representative studies listed in Table 3 had a mean Se of 79%, close to our rate of 72% (Table 2).

Certain patients presenting with chronic PJI require multiple surgeries, which may reduce the Se over time. In contrast, comorbidities such as diabetes may increase the Se. Therefore, it is possible that the pre-test probability differs among patients [26].

AGS afforded good Sp but very low Se (cf. Table 2). However, these findings were not statistically significant given the small number of patients (18 of 168). Moreover, this technique was used only when the ESR was < 5 mm/h or the number of polynuclear leucocytes was< 2000/mm^3^, rendering measurement conditions unfavourable regardless of the method used.

In terms of the utility of hybrid imaging, the addition of SPECT-CT to LS increased the Sp to 80%, supporting what other authors have also found [9–11]. SPECT-CT may be useful for differentiating soft tissue infections from bone infections. Nevertheless, metallic artefacts sometimes limit the ability of SPECT-CT to identify the location of lesions, and tomodensitometry adds to the burden of patient irradiation. We found that the Ses of planar and SPECT-CT imaging were rather similar (68% vs. 67%).

A negative BS indicates a low probability of periprosthetic infection [9, 10, 27]. We found that the NPV was low (50%) (cf. Table 2 and Fig. 1). Selection bias was probably at play; BS was often positive because of mechanical loosening.

LS seems to be more sensitive to TKA than to THA infection; we can offer no obvious explanation for this. Certain factors may increase the Se. These include differences between knee and hip loadings, the surgical technique used (whether cement is employed to fix the prosthesis or not; a cementless prosthesis may be associated with more inflammation), and joint characteristics (the knee is more superficial than the hip, which may be associated with greater predispositions to inflammation and infection).

We found no correlation between the current antibiotic treatment and LS values, as was also true in other studies [11, 26, 28, 29].

To the best of our knowledge, no literature data are available on LLS values in relation to the extent of WBC labelling. According to the Society of Nuclear Medicine, the usual amount of ^99m^Tc-HMPAO-labelled WBC injected into adults is 185–370 MBq [30]. The levels varied among published studies: the lowest value was 185 MBq reported by Fernandez et al. [31], while Pelosi et al. injected 430–600 MBq [24], Filippi et al. 400–555 MBq [19], and Simonsen et al. 628–775 MBq [25]. We were not able to determine the best compromise between Se and irradiation. Moreover, the separation of polynuclear leucocytes from other blood cells was less than perfect. Therefore, somered blood cells may have been labelled, which could reduce the Se of LLS and limit the comparisons made among the various activity groups. However, influence of these labelled red blood cells has not been studied and we did not know if there is a correlation between activity of this extra leucocyte labelling and Se of the scintigraphy.

As FDG accumulates at sites of infection and inflammation, positron emission tomography (PET) has been used to diagnose disorders of the musculoskeletal system, including PJI. However, FDG imaging afforded no significant advantage over LS [5, 14].

Leucocyte uptake around a prosthesis may reflect bone marrow displacement or surgical activation. Therefore, LS has been combined with 99mTc-sulphur colloid. In patients lacking PJI, the bone marrow distributions evident on LS and bone marrow scans are similar. The reported accuracy of the LS/bone marrow scan combination ranged from 86 to 98% [32].

## Conclusion

LLS is a very interesting imaging modality used to explore chronic bone and joint infections. In practice, we found that AGS was less efficient but preferred when LLS was not possible for various reasons. In a selected population, we found that the Se of LS varied by the location of the infection. The current antibiotic treatment, the type of pathogen, and the injected activity had no influence on LS accuracy. Hybrid imaging using SPECT-CT seemed to increase the Sp of LS. Thus, PET/CT imaging may be the next step in leucocyte exploitation. The radiolabel (^18^F) has a short half-life (110 min), and labelled leucocyte PET is thus unsuitable for data acquisition later than 4–6 h after injection. In cases of chronic PJI, delayed imaging is mandatory because of slow leucocyte biokinetics at the infected sites. It will be interesting to follow the contributions made by new radiotracers such as ^64^Cu, the half-life (2.6 days) of which allows radiolabelled antibodies or white blood cells to be tracked.
